# Matrix Metalloproteinase Proteolysis of the Myelin Basic Protein Isoforms Is a Source of Immunogenic Peptides in Autoimmune Multiple Sclerosis

**DOI:** 10.1371/journal.pone.0004952

**Published:** 2009-03-20

**Authors:** Sergey A. Shiryaev, Alexei Y. Savinov, Piotr Cieplak, Boris I. Ratnikov, Khatereh Motamedchaboki, Jeffrey W. Smith, Alex Y. Strongin

**Affiliations:** Inflammatory and Infectious Disease Center, Burnham Institute for Medical Research, La Jolla, California, United States of America; University of Nebraska, United States of America

## Abstract

**Background:**

Matrix metalloproteinases (MMPs) play a significant role in the fragmentation of myelin basic protein (MBP) and demyelination leading to autoimmune multiple sclerosis (MS) and experimental autoimmune encephalomyelitis (EAE). The classic MBP isoforms are predominantly expressed in the oligodendrocytes of the CNS. The splice variants of the single MBP gene (Golli-MBP BG21 and J37) are widely expressed in the neurons and also in the immune cells. The relative contribution of the individual MMPs to the MBP cleavage is not known.

**Methodology/Principal Findings:**

To elucidate which MMP plays the primary role in cleaving MBP, we determined the efficiency of MMP-2, MMP-8, MMP-9, MMP-10, MMP-12, MT1-MMP, MT2-MMP, MT3-MMP, MT4-MMP, MT5-MMP and MT6-MMP in the cleavage of the MBP, BG21 and J37 isoforms in the *in vitro* cleavage reactions followed by mass-spectroscopy analysis of the cleavage fragments. As a result, we identified the MMP cleavage sites and the sequence of the resulting fragments. We determined that MBP, BG21 and J37 are highly sensitive to redundant MMP proteolysis. MT6-MMP (initially called leukolysin), however, was superior over all of the other MMPs in cleaving the MBP isoforms. Using the mixed lymphocyte culture assay, we demonstrated that MT6-MMP proteolysis of the MBP isoforms readily generated, with a near quantitative yield, the immunogenic N-terminal 1–15 MBP peptide. This peptide selectively stimulated the proliferation of the PGPR7.5 T cell clone isolated from mice with EAE and specific for the 1–15 MBP fragment presented in the MHC H-2^U^ context.

**Conclusions/Significance:**

In sum, our biochemical observations led us to hypothesize that MT6-MMP, which is activated by furin and associated with the lipid rafts, plays an important role in MS pathology and that MT6-MMP is a novel and promising drug target in MS especially when compared with other individual MMPs.

## Introduction

Matrix metalloproteinases (MMPs) comprise a family of 24 enzymes that are expressed by many cell types, especially in malignancy. Membrane-tethered MMPs (MT-MMPs) are distinguished from soluble MMPs by the additional transmembrane and cytoplasmic domains (MT1-3 MMP and MT5-MMP). In contrast to these four MT-MMPs, MT4-MMP and MT6-MMP are attached to the cell membrane *via* a glycosylphosphatidyl inositol (GPI) anchor. MMPs cleave the components of the extracellular matrix as well as multiple growth factors, cytokines and cell-surface receptors. MMPs are synthesized as latent zymogens. To become proteolytically active, MMPs require proteolytic removal of the N-terminal prodomain. MMP-11, MMP-28 and several MT-MMPs with the motif RXK/RR in their propeptides are activated by furin [1], [2].

Multiple sclerosis (MS) is a disease of the CNS with autoimmune etiology. Experimental autoimmune encephalomyelitis (EAE), an inducible disease in laboratory animals, is a widely accepted model of MS. EAE is induced by autoreactive CD4^+^ T cells specific for myelin antigens. Myelin proteins including proteolipid protein, myelin oligodendrocyte glycoprotein and especially myelin basic protein (MBP) are candidate autoantigens in MS. MS is characterized by multiple regions of focal myelin loss (lesions) and infiltration of macrophages and lymphocytes in the lesions [3]–[7]. The demyelination process is manifested as a result of interactions among encephalitogenic, regulatory and accessory cell populations and factors, including MMPs produced by these cells. In MS, MMPs could be responsible for the influx of inflammatory mononuclear cells into the CNS, contribute to myelin destruction and affect the integrity of the blood-brain barrier.

Evidence suggests that multiple MMPs cleave MBP and generate immunogenic peptides and that EAE can be induced by immunization with MBP or its 1–15, 68–86, 83–99, 84–104 and 87–99 fragments [8]–[11]. Immunoreactive MBP fragments appear in the cerebrospinal fluid in MS patients [12], [13]. MBP and its Golli splice variants are transcribed from a single gene in humans and mice [14]. This gene contains three transcription start sites. The first start site is responsible for the synthesis of the Golli BG21 and J37 isoforms. The MBPs are expressed from the two downstream transcription start sites [15]. There are five classic MBP splice products and three Golli splice products. Of the Golli splice forms only BG21 and J37 contain MBP sequence. BG21 and J37 are expressed in the CNS and peripheral nervous system and in immune cells [14], [16]–[18]. The “classic” MBP is predominantly expressed in the nervous system [15]. The Golli-MBP isoforms play an incompletely understood role in MS [19], [20].

To determine the identity of MMPs which are efficient in the MBP cleavage and which can contribute to the demyelination processes, we focused our studies on the individual MMPs which represent the major MMP groups including gelatinases (MMP-2 and MMP-9), the simple hemopexin MMPs (MMP-8, -10 and -12), the membrane associated MT-MMPs (MT1-3 and MT5-MMP) and the GPI-linked MMPs (MT4-MMP and MT6-MMP). We determined both the efficiency of the individual MMPs in the cleavage of the MBP isoforms and the precise sequence of the cleavage sites. We demonstrated that MT6-MMP proteolysis of the MBP isoforms generated the highly immunogenic N-terminal MBP peptide which efficiently stimulated the proliferation of the specific T cell clone isolated from mice with EAE. Based on our data, we now suspect that by cleaving the MBP and Golli-MBP isoforms MT6-MMP plays a significant role in the origin of MS in humans.

## Results

### Isolation of the recombinant constructs

**Figure 1:**
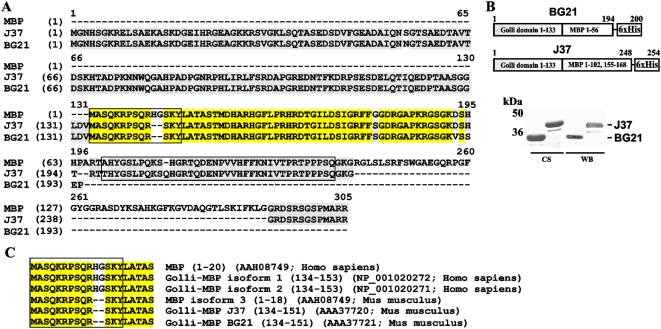
The sequence of the MBP isoforms and the purified BG21 and J37. A, Sequence alignment of murine Golli-MBP BG21 (GenBank #AAA37721), murine Golli-MBP J37 (GenBank #AAA37720) and human MBP (18.5 kDa; GenBank AAH08749). The immunogenic fragments are boxed. Identical residues between two proteins are in grey. Identical residues among three proteins are in yellow. B. Constructs and purification of BG21 and J37. Upper panel - BG21 includes the 1–133 Golli domain and the 1–56 MBP domain. J37 includes the 1–133 Golli domain and the 1–102 and 155–168 fragments of the MBP domain. The constructs were C-terminally tagged with a Hisx6 tag. Bottom panel – purified BG21 and J37. CS, Coomassie staining. WB, Western blotting with a rabbit polyclonal antibody raised against amino acids 1–133 of the Golli domain [21], [49]. C, Sequence alignment of the human and murine MBP, BG21 and J37 isoforms. Identical residue positions are in yellow. Immunogenic peptide sequences are boxed.

The murine Golli-MBP BG21 and J37 constructs were C-terminally tagged with a Hisx6 tag (Fig. 1). The constructs were expressed in *E. coli* and purified using metal-chelating chromatography. The purified BG21 and J37 samples were readily recognized by a rabbit polyclonal antibody raised against amino acids 1–133 of the Golli domain [21] (Fig. 1).

### *In vitro* cleavage of MBP, BG21 and J37 by MMPs

**Figure 2:**
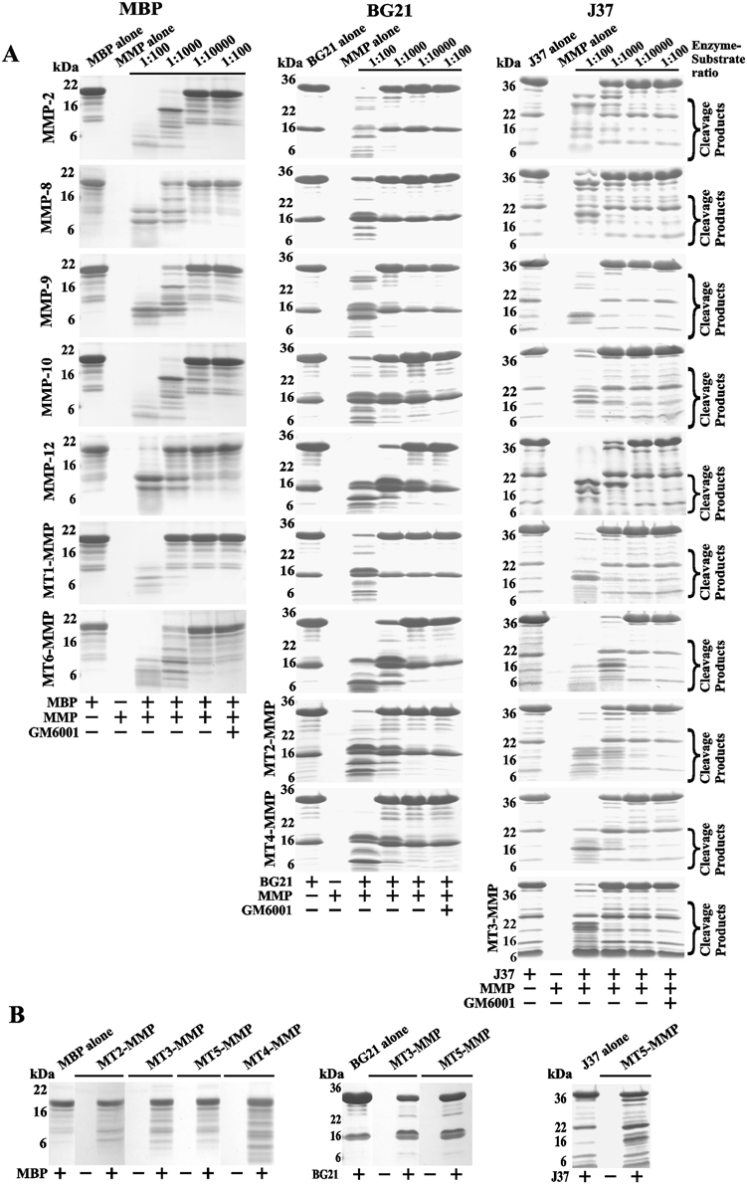
MMPs cleave MBP, BG21 and J37. A, Gel-electrophoresis of the digest samples. MBP, BG21 and J37 were incubated alone for 60 min at 37°C or co-incubated with the individual MMPs at the indicated enzyme-substrate molar ratio. Where indicated, GM6001 was added to the reactions to block MMPs. B, The MBP isoforms are inefficiently cleaved by certain MMPs. MBP, BG21 and J37 were each co-incubated for 60 min at 37°C with the indicated MMPs at a 1∶100 enzyme-substrate ratio.

MBP was co-incubated for 1 h at 37°C with the individual MMPs. The digests were separated by SDS-PAGE (Fig. 2). Where indicated, the samples included GM6001 (a potent, broad-range inhibitor of MMPs). MBP was sensitive to proteolysis by many individual MMPs. MMP-2, MMP-10 and MT6-MMP, however, were the most efficient in cleaving MBP while MT2-MMP, MT3-MMP, MT4-MMP and MT5-MMP were the least efficient. Similarly, MMP-12 and especially MT6-MMP were the most efficient in the proteolysis of the BG21 and J37 constructs while MT5-MMP was the least efficient (Fig. 2). We determined that MT6-MMP was the most efficient among all of the individual MMPs we tested for cleaving the MBP, BG21 and J37 constructs.

### MS analysis of the digest reactions

**Figure 3:**
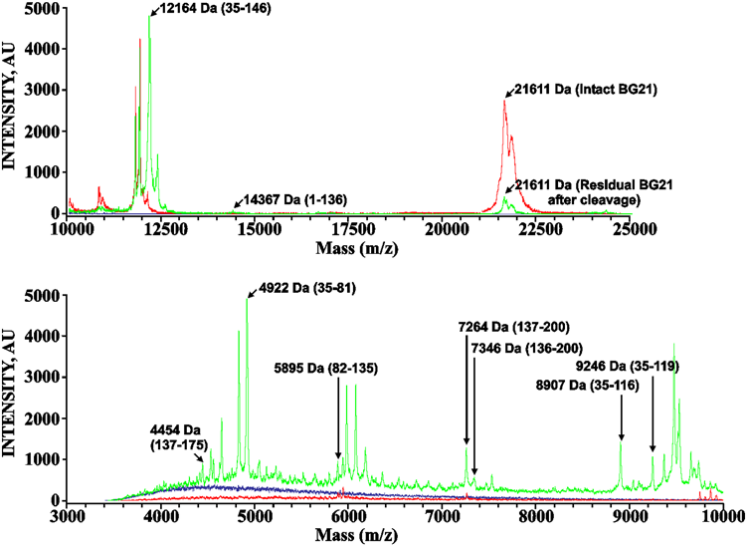
Representative MALDI-TOF MS spectra of the BG21 sample. BG21 (10 µM) was co-incubated for 60 min at 37°C with MT6-MMP (0.01 µM). The resulting peptides were analyzed using a Bruker Daltonics Autoflex II MALDI TOF TOF mass spectrometer to determine their molecular mass. The high and low molecular mass fragments are shown in the top and bottom panels, respectively. The intact and the digested BG21 samples are in green and red, respectively. The buffer alone, blue line. The numbers in the parentheses show the numbering of the peptide in the BG21 sequence.

**Figure 4:**
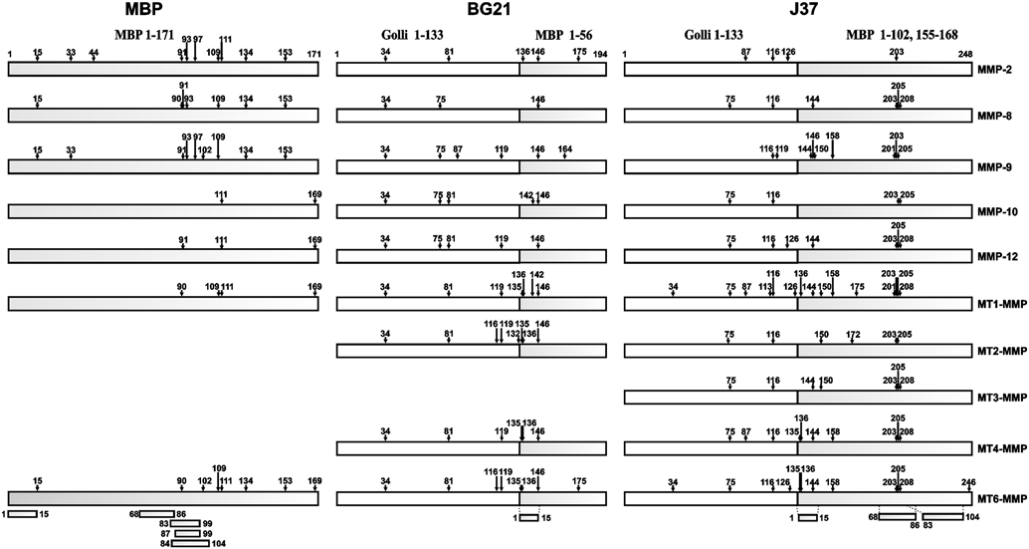
The cleavage map of MBP, BG21 and J37. The numbers indicate the position of the cleavage sites. The immunogenoic regions are shown at the bottom of the panel using the MBP residue numbering.

The MBP, BG21 and J37 constructs were subjected to proteolysis by the individual MMPs. The mass of the digest peptides was then determined using MALDI-TOF MS. The results of a representative MS analysis of MT6-MMP proteolysis of BG21 are shown in Fig. 3. In our analyses, we tried to identify the most representative cleavage fragments rather than trying to determine the identity of each peptide in the samples. To map the experimental cleavage sites we used a cleavage prediction program that predicted both the MMP cleavage sites and the size of the digest fragments. These predictions aided in the precise identification of the size and the sequence of the cleavage fragments. The selected partial data are presented in Tables S1, S2, S3. The cleavage maps of the MBP, BG21 and J37 isoforms are schematically presented in Fig. 4.

According to our cleavage data, multiple MMPs including MT6-MMP were capable of readily generating the C-terminal and especially N-terminal digest peptides of the MBP portion of the constructs. These peptides were highly similar to the known immunogenic sequence regions of MBP which were capable of causing EAE in animal models [9]–[11]. The peptide that corresponded to the known immunogenic region 68–86 from the central part of MBP cannot be generated by MMPs because it was cleaved as a result of MMP proteolysis of the MBP, BG21 and J37 isoforms. Generally, because of the cleavage preference redundancy among MMPs, multiple individual MMPs are capable of generating, albeit with widely varying kinetics, similar cleavage peptide sequences as a result of the proteolysis of the MBP and Golli-MBP isoforms.

The sequence of the 1–15 region is conserved in the human and murine MBP and Golli-MBP isoforms and only a two-residue deletion discriminates the murine and human MBP proteins. As a result of MMP proteolysis of BG21, only a single known, 1–15, immunogene could be generated while the cleavage of J37 could generate both the N- and the C-terminal immunogenic sequences. Overall, MT6-MMP was the most potent in cleaving the MBP, BG21 and J37 isoforms and in generating the N-terminal digest product that corresponded to the known 1–15 immunogenic region of MBP.

### The cleavage of the synthetic peptides by MMPs

To corroborate the MS data and to confirm that MMPs cleave the SKY↓LAT sequence in the MBP isoforms and generate the immunogenic product that corresponds to the 1–15 sequence of MBP, we subjected the synthetic peptides ASQKRPSQRHGSKYLATAS and ASQKRPSQRSKYLATAS (1 µg each, ∼50 µM) to proteolysis by MMP-2, MMP-8, MMP-9 and MT6-MMP (25 nM each). The first peptide corresponded to the 2–20 sequence of human MBP while the second peptide corresponded to the 2–18 sequence of murine MBP. The digests were then analyzed by MALDI-TOF MS. MMP-10 which, according to our data, could not cleave the SKY↓LAT was used as a control. Consistent with our earlier results, MMP-2, MMP-8, MMP-9 and MT6-MMP but not MMP-10 cleaved the peptides and generated the ASQKRPSQRHGSKY and ASQKRPSQRSKY N-terminal cleavage products of the expected molecular mass (Table 1).

**Table 1:** Cleavage of the murine (2–18) and human (2–20) MBP peptides by MMPs.

	Human MBP 2–20 ASQKRPSQRHGSKYLATAS		Murine MBP 2–18 ASQKRPSQRSKYLATAS	
	Molecular mass, Da		Molecular mass, Da	
	Calculated	Measured	Calculated	Measured
Intact peptide	2073.28	2071.90	1879.09	1877.60
Digest fragment	ASQKRPSQRHGSKY		ASQKRPSQRSKY	
MMP-2	1629.78	1628.54	1435.58	1434.98
MMP-8		1628.56		1441.21
MMP-9		1628.40		1435.02
MT6-MMP		1629.83		1435.92
MMP-10		No cleavage		No cleavage

### Activation T cell clone specific to the murine MBP 1–15 fragment

**Figure 5:**
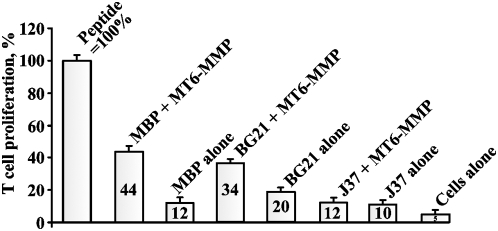
MT6-MMP proteolysis of MBP generates highly specific immunogenic peptides which efficiently stimulate the proliferation of the specific T cell clone. MBP, BG21 and J37 (5 µM each) were cleaved by MT6-MMP (an enzyme-substrate ratio of 1∶100). The irradiated splenocytes from B10.PL mice were co-incubated with the digest reactions. The CD4^+^ T cells (clone PGPR7.5) specific for the murine 1–15 MBP peptide presented in the MHC H-2^U^ context were then added to the reactions. H^3^-thymidine was then added to the cells. The incorporation of the label into the T cells was measured by liquid scintillation counting. MBP alone, BG21 alone and J37 alone - intact MBP, BG21 and J37 (5 µM each) were added to the cells. Cells alone, no peptide. The 1–15 ASQKRPSQRSKYLATAS MBP peptide (5 µM) was used as a control ( = 100%). The numbers show the percentage relative to the peptide control.

To directly show that MT6-MMP proteolysis generates the immunogenic 1–15 peptide, we used the digests to stimulate, in the mixed lymphocyte cultures, the proliferation of the murine T cells clone which is specific to the 1–15 fragment of MBP. The PGPR7.5 clone which is specific for the murine MBP 1–15 peptide presented in the MHC H-2^U^ context was isolated from EAE mice [22]. MBP, BG21 and J37 (5 µM each) were cleaved by MT6-MMP. The irradiated splenocytes from B10.PL mice were co-incubated with the digests. The PGPR7.5 T cells and H^3^-thymidine were then added to the reactions. The incorporation of the label into the T cells was measured by liquid scintillation counting. The digest products were internalized and presented by the splenocytes and then the presented peptides stimulated the proliferation of the specific PGPR7.5 T cells. As a control, we used the synthetic ASQKRPSQRSKYLATAS peptide (5 µM) that corresponded to the 2–18 sequence of murine MBP and the stimulatory effect of which we took as 100%. The MT6-MMP digest of MBP resulted in a 44% level of proliferation of PGPR7.5 T cells compared to the equimolar amount of the ASQKRPSQRSKYLATAS peptide, thus suggesting a high yield of the specific immunogenic fragment in the digest (Fig. 5).

A comparable level (34%) of stimulation of the specific PGPR7.5 T cell clone was observed when the MT6-MMP digest of BG21 was used in the mixed lymphocyte culture assay. This lower yield could be explained by the generation of the peptides (S^136^QKRPSQRSKY^146^ and Q^137^KRPSQRSKY^146^), which were truncated from the N-end when compared to the N-terminal 1–15 ASQKRPSQRHGSKY MBP peptide. Both because of the cleavage preference of MT6-MMP and the peptide sequence of J37, overly short peptides (S^136^QKRPSQRS^144^ and Q^137^KRPSQRS^144^) which are truncated from both the N- and C-ends, will be predominantly generated as a result of the J37 cleavage. It is not surprising that these peptides were not as efficient in the mixed lymphocyte culture assay as the full-length 1–15 MBP peptide.

## Discussion

Several MMP family members contribute to pathology in MS and EAE [23]–[27]. The existing data suggest that there are links among MMP proteolysis of MBP, demyelination and MS [28]. Theses events cause an MBP deficiency, myelin sheath destruction and axon degeneration leading to MS [24]. When fragmented, MBP generates several immunogenic peptides which are potent immunogens [5], [6], [29]–[31]. EAE is an animal model of autoimmune MS-like diseases and it is induced by immunization with MBP fragments to induce autoimmune demyelinating EAE in animals. Several fragments of MBP including 1–15, 68–86, 83–99, 84–104 and 87–99 are known to generate EAE efficiently [9]–[11]. Multiple MMP types may be involved in the cleavage of neuronal MBP in both EAE mice and MS patients [24].

The expression of the classic MBP transcripts is largely restricted to myelin-forming cells. In turn, the splice variants of MBP called Golli-MBP BG21 and J37 are expressed in the thymus, spleen, and lymph nodes [15], [32]. In addition to lymphoid cells, the Golli-MBP isoforms are present in the myeloid lineage cells, including macrophages, dendritic cells and granulocytes, as well as in megakaryocytes and erythroblasts. Because of the presence of the common exons in MBP, BG21 and J37, their fragmentation can generate similar immunogenic peptides including the fragment from the 1–9 and 1–15 immunogenic region and a source of a dominant T cell clonotype in EAE [33].

Our model *in vitro* cleavage experiments in which we used multiple individual MMPs and which were followed by MALDI-TOF MS analysis of the MBP, BG21 and J37 fragments demonstrated that multiple individual MMPs including MMP-2, MMP-8, MMP-9, MMP-10, MMP-12, MT1-MMP and MT6-MMP were capable of efficiently cleaving the MBP, BG21 and J37 isoforms. The GPI-linked MT6-MMP/MMP-25, however, was the most efficient in both cleaving these isoforms and generating the immunogenic 1–15 MBP fragment. The specificity of MT6-MMP proteolysis of the MBP, BG21 and J37 proteins and the extremely high, near quantitative, yield of the 1–15 fragment was confirmed by using the mixed lymphocyte cultures in which proliferation of the PGPR7.5 T cell clone specific for the 1–15 fragment of MBP presented in the MHC H-2^U^ context was stimulated by the digest samples and the level of stimulation was compared to that generated by the equimolar amount of the synthetic 1–15 ASQKRPSQRSKYLATAS MBP peptide. Consistent with our results, MT1-MMP and MT6-MMP are upregulated in the spinal cord of SJL mice with severe EAE induced by adoptive transfer of myelin basic protein-reactive T cells while the other four MT-MMPs are down-regulated [34].

MT6-MMP is a distant relative of MT1-MMP, an archetype membrane-tethered MMP. MT6-MMP is an insufficiently studied, GPI-linked, lipid raft-associated cell surface MMP with an incompletely understood regulation and function [35]–[38]. Among MMPs, the expression of MT6-MMP (initially called leukolysin) is most selectively linked to the leukocyte lineage cells [39]–[41] and up-regulated in certain cancer types including brain tumors [37], [41]. It should be noted that BG21 relocates to the caveolae-enriched lipid rafts upon phorbol ester stimulation of T cells leading to the activation of protein kinase C [42]. The presence of both BG21 and GPI-linked MT6-MMP in the lipid rafts increases the opportunity for the selective MT6-MMP proteolysis of the Golli proteins in the stimulated immune system cells.

Overall, our results on the redundant role of multiple individual MMPs in the destruction of MBP correlate well with the observations by many authors who have suggested the important role MMPs play in the cleavage of MBP, leading to the generation of immunogenic fragments and demyelination in both EAE and human MS [15], [24], [43], [44]. Our results support and extend these earlier observations. Our combined biochemical observations have led us to hypothesize that MT6-MMP plays an especially significant role in the proteolytic pathway to MS and, therefore, may represent a novel and promising drug target. We now believe that the proteolytic pathway leading to MS involves the MT6-MMP proteolysis of the Golli-MBP isoforms in the immune system cells followed by the stimulation of the specific autoimmune T cell clones which then home through the disrupted blood-brain barrier to the brain and recognize neuronal MBP. In the brain, these autoimmune T cells can cause inflammation leading to further upregulation of the activity of multiple MMPs and the massive cleavage of MBP in the brain resulting in demyelination and MS. Our current on-going experiments support this hypothesis (manuscript in preparation).

## Materials and Methods

### Reagents

All reagents unless otherwise indicated were from Sigma. A hydroxamate inhibitor of MMPs (GM6001) was from Chemicon. A hydroxamate inhibitor of MMPs AG3340 was kindly provided by Dr. Peter Baciu (Allergan, Irvine, CA). MBP (18.5 kDa isoform, GenBank #AAH08749) was purchased from Biodesign. The CD^4+^ T cells specific to murine MBP 1–15 fragment were kindly provided by Dr. Vipin Kumar (Torrey Pines Institute for Molecular Studies, San Diego, CA). The ASQKRPSQRHGSKYLATAS and ASQKRPSQRSKYLATAS peptides, which corresponded to the sequence 2–20 of human MBP and 135–153 of BG21 and J37, and to the sequence 2–18 of murine MBP and 135–151 of BG21 and J37, respectively, were synthesized by GenScript.

### Expression and purification of Golli-MBP BG21 and J37

The plasmids pET22B-BG21 and pET22B-J37 expressing Golli-MBP BG21 (GenBank #AAA37721) and Golli-MBP J37 (GenBank #AAA37720) were constructed earlier [42]. The J37 and BG21 constructs were C-terminally tagged with a 6xHis tag. *E. coli* BL21 (DE3)-Codon Plus-RIPL cells (Stratagene) were transformed using the plasmids. The expression of the J37 and BG21 constructs was induced by 1 mM isopropyl β-D-thiogalactoside for 6 h. The BG21 and J37 proteins were isolated from the soluble fraction of E. coli cells on a HiTrap Co^2+^-chelating Sepharose FastFlow column (GE Healthcare). The constructs were eluted with a 0–500 mM gradient of imidazole concentrations, concentrated using a 5 kDa-cutoff concentrator (Millipore) and dialyzed against 10 mM Tris-HCl, pH 8.0, containing 200 mM NaCl.

### In vitro cleavage reactions and mass-spectrometry analysis

The ASQKRPSQRHGSKYLATAS and ASQKRPSQRSKYLATAS peptides (1 µg; ∼50 µM each) were co-incubated with the individual MMPs (25 nM) for 1 h at 37°C in 50 mM HEPES, pH 6.8, supplemented with 10 mM CaCl_2_, 0.5 mM MgCl_2_ and 50 mM ZnCl_2_. The mass of the digested peptides was determined by Matrix-Assisted Laser Desorption Ionization-Time of Flight Mass Spectrometry (MALDI-TOF MS) using an Autoflex II MALDI TOF/TOF instrument (Bruker Daltonics). For mass spectrometric analysis, equal volumes of a sample and of an α-cyano-4-hydroxy-cinnamic acid (20 mg/ml) in 50% acetonitrile-0.1% trifluoroacetic acid solution were co-crystallized directly on the MALDI target plate and allowed to dry for 5 min.

MBP (4 µg; ∼10 µM) and Golli-MBP BG21 (4 µg, ∼10 µM) and J37 (5.5 µg, ∼10 µM) were co-incubated with the individual MMPs (1–100 nM) in 50 mM HEPES, pH 6.8, supplemented with 10 mM CaCl_2_ and 50 µM ZnCl_2_, for 1 h at 37°C. Where indicated, GM6001 (2.5 mM) was added to the reactions to inhibit MMPs. The cleavage was stopped using a 5xSDS sample buffer. The digest samples were analyzed by SDS-PAGE and by MALDI-TOF MS. For mass spectrometry analysis, the reactions were cooled on ice and equal volumes of a sample and of a sinapic acid (20 mg/ml) in 50% acetonitrile-0.1% trifluoroacetic acid solution were co-crystallized directly on the MALDI target plate and allowed to dry for 5 min. Mass spectra were processed with FlexAnalysis 2.4. The singly charged cleavage products, which were observed only in the cleavage reactions but not in the controls, were recorded and processed further.

### Expression and purification of MMPs

The individual catalytic domains of MMP-10 and MMP-12 were purchased from Biomol. The individual catalytic domains of MMP-2, -8, -9, and MT1-MMP, MT2-MMP, MT3-MMP, MT4-MMP, MT5-MMP and MT6-MMP were expressed in *E. coli*, purified from the inclusion bodies using metal-chelating chromatography and refolded to restore their native conformation [45]. The refolded MMPs were used immediately in activity assays. The concentration of the catalytically active MMPs was measured using a fluorescent assay by titration against a standard AG3340 solution of known concentration. (7-methoxycoumarin-4-yl)Acetyl-Pro-Leu-Gly-Leu-(3-[2,4-dinitrophenyl]-L-2,3-diaminopropionyl)-Ala-Arg-NH_2_ (Bachem) was used as a fluorescent substrate. The steady-state rate of the substrate cleavage by MMP was plotted as a function of inhibitor concentration and fitted with the equation V = SA(E_0_−0.5{(E_0_+I+*K_i_*)−[(E_0_+I+*K_i_*)^2^−4E_0_I]^0.5^}), where *V* is the steady-state rate of substrate hydrolysis, *SA* is specific activity (rate per unit of enzyme concentration), *E_0_* is enzyme concentration. *I* is inhibitor concentration, and *K_i_* is the dissociation constant of the enzyme-inhibitor complex [46].

### Prediction and ranking of the cleavage sites

To predict the cleavage sites for the individual MMPs in the sequence of MBP and Golli-MPBs BG21 and J37 we used a specialized computer program we had developed. The program determines the contribution of each amino acid residue at each of the P3-P2′ positions to the efficiency of the protein proteolysis by a proteinase and assigns a numerical score to every peptide bond in the protein sequence. The score is based on the Positional Weight Matrix (PWM) approach we developed for the individual MMPs using the high volume data from the substrate phage library cleavage. The elements of the PWM define the probability of the presence of each amino acid type at the P3 to P2′ sub-site position of the substrate relative to the cleavage-resistant substrates. The PWM score is a sum of the log_2_ elements (log-odds) of the P3 to P2′ positions (eq.1–2).
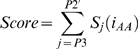
(1)

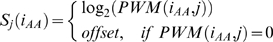
(2)where *j* is the j-th position near the cleavage site and *i_AA_* is the amino acid type. The *offset* and *threshold* values are specific for each MMP and they were determined using the 10-fold cross-validation test. A peptide bond is considered to be cleavable if the score value exceeds the threshold value. The *offset* is the log_2_ value of the score when the PWM element equals to zero. The data were filtered using the Jnet secondary structure prediction software [47] and the Disopred2 disordered region prediction software [48]. Only those potential cleavage sites which were predicted by both programs to be at the high, 5–9, confidence levels to be localized in the unstructured and disordered regions were considered further.

### Stimulation of the murine T cell clone PGPR7.5 specific to the 1–15 MBP fragment

Prior to the assay, the *E. coli*-derived BG21 and J37 samples were purified using an Affinity Pak Detoxi-Gel kit (Thermo Scientific) to remove LPS. MBP, Golli-MBP BG21 and Golli-MBP J37 (5 µM each) were cleaved for 1 h at 37°C by MT6-MMP (0.05 µM; an enzyme-substrate ratio of 1∶100). The irradiated splenocytes from B10.PL mice (1×10^6^) were co-incubated for 1 h with the digest reactions. As controls, intact MBP and the murine 2–18 MBP/135–151 Golli-MBP peptide ASQKRPSQRSKYLATAS (5 µM each) were used. The CD4^+^ T cells (5×10^5^; clone PGPR7.5) specific for murine 1–15 peptide presented in the MHC H-2^U^ context were then added to the reactions for 72 h. H^3^-thymidine (1 µ Ci) was then added to the cells for 14 h. The incorporation of the label into the T cells was measured by liquid scintillation counting.

## Supporting Information

Table S1 MMP proteolysis of MBP and a MALDI-TOF MS analysis of the digest fragments. The arrows indicate the positions of the scissile bonds. The numbering starts from the N-terminal methionine.
(0.10 MB DOC)

Table S2 MMP proteolysis of Golli-MBP BG21 and a MALDI-TOF MS analysis of the digest fragments. The arrows indicate the positions of the scissile bonds. The numbering starts from the N-terminal methionine.
(0.13 MB DOC)

Table S3 MMP proteolysis of Golli-MBP J37 and a MALDI-TOF MS analysis of the digest fragments. The arrows indicate the positions of the scissile bonds. The numbering starts from the N-terminal methionine.
(0.14 MB DOC)
