# Supplementary material for: Matrix Metalloproteinase Proteolysis of the Myelin Basic Protein Isoforms Is a Source of Immunogenic Peptides in Autoimmune Multiple Sclerosis
Source: PLoS One. 2009 Mar 20;4(3):e4952. doi: 10.1371/journal.pone.0004952 (PMC2654159; doi:10.1371/journal.pone.0004952)
Supplement: Table S1 — MMP proteolysis of MBP and a MALDI-TOF MS analysis of the digest fragments. The arrows indicate the positions of the scissile bonds. The numbering starts from the N-terminal methionine. (0.10 MB DOC) [file pone.0004952.s001.doc]

**Supplemental Table 1. MMP proteolysis of MBP and a MALDI-TOF MS analysis of the digest fragments.** The arrows indicate the positions of the scissile bonds. The numbering starts from the N-terminal methionine.

1)

| MMP-2 fragments | | | |
| --- | --- | --- | --- |
| asqkrpsqr^10^ hgsky↓latas^20^ tmdharhgfl^30^ prh↓rdtgild^40^ sigr↓ffggdr^50^  gapkrgsgkd^60^ shhpartahy^70^ gslpqkshgr^80^ tqdenpvvhf^90^ f↓kn↓ivtp↓rtp^100^  ppsqgkgrg↓l^110^ s↓lsrfswgae^120^ gqrpgfgygg^130^ rasd↓yksahk^140^ gfkgvdaqgt^150^  lsk↓ifklggr^160^ dsrsgspmar^170^ r^171^ | | | |
| Peptide sequences | | Molecular mass, Da | |
|  |  | Calculated | Measured |
| 1 | 2-134 | 14482 | 14498 |
| 2 | 16-109 | 10229 | 10240 |
| 3 | 16-91 | 8357 | 8356 |
| 4 | 45-111 | 7245 | 7249 |
| 5 | 34-97 | 7010 | 7018 |
| 6 | 94-153 | 6293 | 6284 |

2)

| MMP-8 fragments | | | |
| --- | --- | --- | --- |
| asqkrpsqr^10^ hgsky↓latas^20^ tmdharhgfl^30^ prhrdtgild^40^ sigrffggdr^50^  gapkrgsgkd^60^ shhpartahy^70^ gslpqkshgr^80^ tqdenpvvhf↓^90^ f↓kn↓ivtprtp^100^  ppsqgkgrg↓l^110^ slsrfswgae^120^ gqrpgfgygg^130^ rasd↓yksahk^140^ gfkgvdaqgt^150^  lsk↓ifklggr^160^ dsrsgspmar^170^ r^171^ | | | |
| Peptide sequences | | Molecular mass, Da | |
|  |  | Calculated | Measured |
| 1 | 2-134 | 14482 | 14487 |
| 2 | 16-109 | 10229 | 10243 |
| 3 | 91-171 | 8656 | 8640 |
| 4 | 16-91 | 8357 | 8350 |
| 5 | 94-153 | 6293 | 6284 |

3)

| MMP-9 fragments | | | |
| --- | --- | --- | --- |
| asqkrpsqr^10^ hgsky↓latas^20^ tmdharhgfl^30^ prh↓rdtgild^40^ sigrffggdr^50^  gapkrgsgkd^60^ shhpartahy^70^ gslpqkshgr^80^ tqdenpvvhf^90^ f↓kn↓ivtp↓rtp^100^  pp↓sqgkgrgl^110^ slsrfswgae^120^ gqrpgfgygg^130^ rasd↓yksahk^140^ gfkgvdaqgt^150^  lsk↓ifklggr^160^ dsrsgspmar^170^ r^171^ | | | |
| Peptide sequences | | Molecular mass, Da | |
|  |  | Calculated | Measured |
| 1 | 2-134 | 14482 | 14498 |
| 2 | 16-109 | 10229 | 10247 |
| 3 | 16-91 | 8357 | 8356 |
| 4 | 103-171 | 7307 | 7296 |
| 5 | 34-97 | 7010 | 7018 |
| 6 | 94-153 | 6293 | 6284 |

4)

| MMP-10 fragments | | | |
| --- | --- | --- | --- |
| asqkrpsqr^10^ hgskylatas^20^  tmdharhgfl^30^ prhrdtgild^40^ sigrffggdr^50^  gapkrgsgkd^60^ shhpartahy^70^ gslpqkshgr^80^ tqdenpvvhf^90^ fknivtprtp^100^  ppsqgkgrgl^110^ s↓lsrfswgae^120^ gqrpgfgygg^130^ rasdyksahk^140^ gfkgvdaqgt^150^  lskifklggr^160^ dsrsgspma↓r^170^ r^171^ | | | |
| Peptide sequences | | Molecular mass, Da | |
|  |  | Calculated | Measured |
| 1 | 112-171 | 6436 | 6416 |
| 2 | 112-169 | 6124 | 6105 |

5)

| MMP-12 fragments | | | |
| --- | --- | --- | --- |
| asqkrpsqr^10^ hgskylatas^20^  tmdharhgfl^30^ prhrdtgild^40^ sigrffggdr^50^  gapkrgsgkd^60^ shhpartahy^70^ gslpqkshgr^80^ tqdenpvvhf↓^90^ fknivtprtp^100^  ppsqgkgrgl^110^ s↓lsrfswgae^120^ gqrpgfgygg^130^ rasdyksahk^140^ gfkgvdaqgt^150^  lskifklggr^160^ dsrsgspma↓r^170^ r^171^ | | | |
| Peptide sequences | | Molecular mass, Da | |
|  |  | Calculated | Measured |
| 1 | 91-171 | 8656 | 8638 |
| 2 | 91-169 | 8343 | 8332 |
| 3 | 112-171 | 6436 | 6416 |
| 4 | 112-169 | 6124 | 6103 |

6)

| MT1-MMP fragments | | | |
| --- | --- | --- | --- |
| asqkrpsqr^10^ hgskylatas^20^ tmdharhgfl^30^ prhrdtgild^40^ sigrffggdr^50^  gapkrgsgkd^60^ shhpartahy^70^ gslpqkshgr^80^ tqdenpvvhf↓^90^ fknivtprtp^100^  ppsqgkgrg↓l^110^ s↓lsrfswgae^120^ gqrpgfgygg^130^ rasdyksahk^140^ gfkgvdaqgt^150^  lskifklggr^160^ dsrsgspma↓r^170^ r^171^ | | | |
| Peptide sequences | | Molecular mass, Da | |
|  |  | Calculated | Measured |
| 1 | 2-90 | 9822 | 9830 |
| 2 | 91-171 | 8656 | 8654 |
| 3 | 91-169 | 8343 | 8345 |
| 4 | 110-171 | 6636 | 6614 |
| 5 | 112-171 | 6436 | 6415 |
| 6 | 110-169 | 6324 | 6304 |
| 7 | 112-169 | 6124 | 6103 |

7)

| MT6-MMP fragments | | | |
| --- | --- | --- | --- |
| asqkrpsqr^10^ hgsky↓latas^20^ tmdharhgfl^30^ prhrdtgild^40^ sigrffggdr^50^  gapkrgsgkd^60^ shhpartahy^70^ gslpqkshgr^80^ tqdenpvvhf↓^90^ fknivtprtp^100^  pp↓sqgkgrg↓l^110^ s↓lsrfswgae^120^ gqrpgfgygg^130^ rasd↓yksahk^140^ gfkgvdaqgt^150^  lsk↓ifklggr^160^ dsrsgspma↓r^170^ r^171^ | | | |
| Peptide sequences | | Molecular mass, Da | |
|  |  | Calculated | Measured |
| 1 | 2-134 | 14482 | 14498 |
| 2 | 16-109 | 10229 | 10228 |
| 3 | 2-90 | 9822 | 9830 |
| 4 | 91-171 | 8656 | 8641 |
| 5 | 91-169 | 8343 | 8342 |
| 6 | 112-171 | 6436 | 6411 |
| 7 | 112-169 | 6124 | 6099 |
| 8 | 103-153 | 5334 | 5332 |
