# Supplementary material for: Matrix Metalloproteinase Proteolysis of the Myelin Basic Protein Isoforms Is a Source of Immunogenic Peptides in Autoimmune Multiple Sclerosis
Source: PLoS One. 2009 Mar 20;4(3):e4952. doi: 10.1371/journal.pone.0004952 (PMC2654159; doi:10.1371/journal.pone.0004952)
Supplement: Table S2 — MMP proteolysis of Golli-MBP BG21 and a MALDI-TOF MS analysis of the digest fragments. The arrows indicate the positions of the scissile bonds. The numbering starts from the N-terminal methionine. (0.13 MB DOC) [file pone.0004952.s002.doc]

**Supplemental Table 2. MMP proteolysis of Golli-MBP BG21 and a MALDI-TOF MS analysis of the digest fragments.** The arrows indicate the positions of the scissile bonds. The numbering starts from the N-terminal methionine.

1)

| MMP-2 fragments | | | |
| --- | --- | --- | --- |
| MGNHSGKREL^10^  SAEKASKDGE^20^ IHRGEAGKKR^30^  SVGK↓LSQTAS^40^  EDSDVFGEAD^50^  AIQNNGTSAE^60^  DTAVTDSKHT^70^ ADPKNNWQGA^80^  H↓PADPGNRPH^90^ LIRLFSRDAP^100^  GREDNTFKDR^110^ PSESDELQTI^120^ QEDPTAASGG^130^  LDVMAS↓QKRP^140^ SQRSKY↓LATA^150^  STMDHARHGF^160^ LPRHRDTGIL^170^ DSIGR↓FFSGD^180^ RGAPKRGSGK^190^ VSLEHHHHHH^200^ | | | |
| Peptide sequences | | Molecular mass, Da | |
|  |  | Calculated | Measured |
| 1 | 1-175 | 18813 | 18811 |
| 2 | 35-175 | 15328 | 15328 |
| 3 | 1-136 | 14372 | 14372 |
| 4 | 35-146 | 12146 | 12157 |
| 5 | 35-136 | 10887 | 10890 |
| 6 | 35-81 | 4929 | 4925 |

2)

| MMP-8 fragments | | | |
| --- | --- | --- | --- |
| MGNHSGKREL^10^  SAEKASKDGE^20^  IHRGEAGKKR^30^  SVGK↓LSQTAS^40^ EDSDVFGEAD^50^  AIQNNGTSAE^60^  DTAVTDSKHT^70^  ADPKN↓NWQGA^80^ HPADPGNRPH^90^ LIRLFSRDAP^100^  GREDNTFKDR^110^ PSESDELQTI^120^ QEDPTAASGG^130^ LDVMASQKRP^140^ SQRSKY↓LATA^150^  STMDHARHGF^160^ LPRHRDTGIL^170^ DSIGRFFSGD^180^ RGAPKRGSGK^190^ VSLEHHHHHH^200^ | | | |
| Peptide sequences | | Molecular mass, Da | |
|  |  | Calculated | Measured |
| 1 | 35-200 | 18127 | 18118 |
| 2 | 76-200 | 13909 | 13904 |
| 3 | 35-146 | 12146 | 12157 |

3)

| MMP-9 fragments | | | |
| --- | --- | --- | --- |
| MGNHSGKREL^10^  SAEKASKDGE^20^  IHRGEAGKKR^30^ SVGK↓LSQTAS^40^ EDSDVFGEAD^50^ AIQNNGTSAE^60^  DTAVTDSKHT^70^ ADPKN↓NWQGA^80^ HPADPGN↓RPH^90^ LIRLFSRDAP^100^ GREDNTFKDR^110^ PSESDELQT↓I^120^ QEDPTAASGG^130^ LDVMASQKRP^140^ SQRSKY↓LATA^150^ STMDHARHGF^160^ LPRH↓RDTGIL^170^ DSIGRFFSGD^180^ RGAPKRGSGK^190^ VSLEHHHHHH^200^ | | | |
| Peptide sequences | | Molecular mass, Da | |
|  |  | Calculated | Measured |
| 1 | 76-200 | 13909 | 13910 |
| 2 | 35-146 | 12146 | 12165 |
| 3 | 35-119 | 9244 | 9244 |
| 4 | 88-164 | 8682 | 8684 |
| 5 | 1-75 | 7721 | 7726 |

4)

| MMP-10 fragments | | | |
| --- | --- | --- | --- |
| MGNHSGKREL^10^  SAEKASKDGE^20^ IHRGEAGKKR^30^  SVGK↓LSQTAS^40^ EDSDVFGEAD^50^ AIQNNGTSAE^60^  DTAVTDSKHT^70^ ADPKNNWQGA^80^  HPADPGNRPH^90^ LIRLFSRDAP^100^ GREDNTFKDR^110^ PSESDELQTI^120^ QEDPTAASGG^130^ LDVMASQKRP^140^ SQ↓RSKY↓LATA^150^ STMDHARHGF^160^ LPRH↓RDTGIL^170^ DSIGRFFSGD^180^ RGAPKRGSGK^190^ VSLEHHHHHH^200^ | | | |
| Peptide sequences | | Molecular mass, Da | |
|  |  | Calculated | Measured |
| 1 | 35-146 | 12146 | 12161 |
| 2 | 143-200 | 6533 | 6538 |

5)

| MMP-12 fragments | | | |
| --- | --- | --- | --- |
| MGNHSGKREL^10^ SAEKASKDGE^20^ IHRGEAGKKR^30^ SVGK↓LSQTAS^40^ EDSDVFGEAD^50^ AIQNNGTSAE^60^ DTAVTDSKHT^70^ ADPKN↓NWQGA^80^ H↓PADPGNRPH^90^ LIRLFSRDAP^100^ GREDNTFKDR^110^ PSESDELQT↓I^120^ QEDPTAASGG^130^ LDVMASQKRP^140^ SQRSKY↓LATA^150^ STMDHARHGF^160^ LPRHRDTGIL^170^ DSIGRFFSGD^180^ RGAPKRGSGK^190^ VSLEHHHHHH^200^ | | | |
| Peptide sequences | | Molecular mass, Da | |
|  |  | Calculated | Measured |
| 1 | 35-200 | 18127 | 18130 |
| 2 | 76-200 | 13909 | 13906 |
| 3 | 35-146 | 12146 | 12167 |
| 4 | 35-119 | 9244 | 9247 |
| 5 | 35-81 | 4929 | 4924 |

6)

| MT1-MMP fragments | | | |
| --- | --- | --- | --- |
| MGNHSGKREL^10^ SAEKASKDGE^20^ IHRGEAGKKR^30^  SVGK↓LSQTAS^40^ EDSDVFGEAD^50^  AIQNNGTSAE^60^ DTAVTDSKHT^70^ ADPKNNWQGA^80^  H↓PADPGNRPH^90^ LIRLFSRDAP^100^  GREDNTFKDR^110^ PSESDELQT↓I^120^ QEDPTAASGG^130^ LDVMA↓S↓QKRP^140^ SQ↓RSKY↓LATA^150^  STMDHARHGF^160^ LPRHRDTGIL^170^ DSIGRFFSGD^180^ RGAPKRGSGK^190^ VSLEHHHHHH^200^ | | | |
| Peptide sequences | | Molecular mass, Da | |
|  |  | Calculated | Measured |
| 1 | 1-146 | 15630 | 15620 |
| 2 | 1-136 | 14372 | 14367 |
| 3 | 1-135 | 14285 | 14281 |
| 4 | 35-146 | 12146 | 12159 |
| 5 | 35-119 | 9244 | 9241 |
| 6 | 136-200 | 7345 | 7345 |
| 7 | 137-200 | 7258 | 7259 |
| 8 | 143-200 | 6533 | 6536 |
| 9 | 147-200 | 5999 | 6001 |
| 10 | 35-81 | 4929 | 4922 |

7)

| MT2-MMP fragments | | | |
| --- | --- | --- | --- |
| MGNHSGKREL^10^ SAEKASKDGE^20^ IHRGEAGKKR^30^ SVGK↓LSQTAS^40^ EDSDVFGEAD^50^  AIQNNGTSAE^60^ DTAVTDSKHT^70^ ADPKNNWQGA^80^ H↓PADPGNRPH^90^ LIRLFSRDAP^100^  GREDNTFKDR^110^ PSESDE↓LQT↓I^120^ QEDPTAASGG^130^ LD↓VMA↓S↓QKRP^140^ SQRSKY↓LATA^150^  STMDHARHGF^160^ LPRHRDTGIL^170^ DSIGRFFSGD^180^ RGAPKRGSGK^190^ VSLEHHHHHH^200^ | | | |
| Peptide sequences | | Molecular mass, Da | |
|  |  | Calculated | Measured |
| 1 | 1-135 | 14285 | 14279 |
| 2 | 35-146 | 12146 | 12164 |
| 3 | 35-119 | 9244 | 9246 |
| 4 | 35-116 | 8902 | 8904 |
| 5 | 133-200 | 7646 | 7654 |
| 6 | 136-200 | 7345 | 7349 |
| 7 | 137-200 | 7258 | 7264 |
| 8 | 147-200 | 5999 | 6004 |
| 9 | 35-81 | 4929 | 4926 |

8)

| MT3-MMP fragments | | | |
| --- | --- | --- | --- |
| MGNHSGKREL^10^  SAEKASKDGE^20^ IHRGEAGKKR^30^ SVGK↓LSQTAS^40^ EDSDVFGEAD^50^  AIQNNGTSAE^60^  DTAVTDSKHT^70^ ADPKNNWQGA^80^ H↓PADPGNRPH^90^ LIRLFSRDAP^100^  GREDNTFKDR^110^ PSESDELQT↓I^120^ QEDPTAASGG^130^ LDVMA↓S↓QKRP^140^ SQRSKY↓LATA^150^  STMDHARHGF^160^ LPRHRDTGIL^170^ DSIGRFFSGD^180^ RGAPKRGSGK^190^ VSLEHHHHHH^200^ | | | |
| Peptide sequences | | Molecular mass, Da | |
|  |  | Calculated | Measured |
| 1 | 1-136 | 14372 | 14374 |
| 2 | 35-146 | 12146 | 12165 |
| 3 | 35-119 | 9244 | 9250 |
| 4 | 82-135 | 5889 | 5893 |
| 5 | 35-81 | 4929 | 4924 |

9)

| MT6-MMP fragments | | | |
| --- | --- | --- | --- |
| MGNHSGKREL^10^  AEKASKDGE^20^ IHRGEAGKKR^30^ SVGK↓LSQTAS^40^ EDSDVFGEAD^50^  AIQNNGTSAE^60^  DTAVTDSKHT^70^ ADPKNNWQGA^80^ H↓PADPGNRPH^90^ LIRLFSRDAP^100^  GREDNTFKDR^110^ PSESDE↓LQT↓I^120^ QEDPTAASGG^130^ LDVMA↓S↓QKRP^140^ SQRSKY↓LATA^150^  STMDHARHGF^160^ LPRHRDTGIL^170^ DSIGR↓FFSGD^180^ RGAPKRGSGK^190^ VSLEHHHHHH^200^ | | | |
| Peptide sequences | | Molecular mass, Da | |
|  |  | Calculated | Measured |
| 1 | 1-136 | 14372 | 14367 |
| 2 | 35-146 | 12146 | 12164 |
| 3 | 35-119 | 9244 | 9246 |
| 4 | 35-116 | 8902 | 8907 |
| 5 | 136-200 | 7345 | 7346 |
| 6 | 137-200 | 7258 | 7264 |
| 7 | 82-135 | 5889 | 5895 |
| 8 | 35-81 | 4929 | 4922 |
| 9 | 137-175 | 4459 | 4454 |
