# Supplementary material for: Matrix Metalloproteinase Proteolysis of the Myelin Basic Protein Isoforms Is a Source of Immunogenic Peptides in Autoimmune Multiple Sclerosis
Source: PLoS One. 2009 Mar 20;4(3):e4952. doi: 10.1371/journal.pone.0004952 (PMC2654159; doi:10.1371/journal.pone.0004952)
Supplement: Table S3 — MMP proteolysis of Golli-MBP J37 and a MALDI-TOF MS analysis of the digest fragments. The arrows indicate the positions of the scissile bonds. The numbering starts from the N-terminal methionine. (0.14 MB DOC) [file pone.0004952.s003.doc]

**Supplemental Table 3. MMP proteolysis of Golli-MBP J37 and a MALDI-TOF MS analysis of the digest fragments.** The arrows indicate the positions of the scissile bonds. The numbering starts from the N-terminal methionine.

1)

| MMP-2 fragments | | | |
| --- | --- | --- | --- |
| MGNHSGKREL^10^ SAEKASKDGE^20^ IHRGEAGKKR^30^ SVGKLSQTAS^40^ EDSDVFGEAD^50^  AIQNSGTSAE^60^ DTAVTDSKHT^70^ ADPKNNWQGA^80^ HPADPGN↓RPH^90^ LIRLFSRDAP^100^  GREDNTFKDR^110^ PSESDE↓LQTI^120^ QEDPTA↓ASGG^130^ LDVMASQKRP^140^ SQRSKYLATA^150^  STMDHARHGF^160^ LPRHRDTGIL^170^ DSIGRFFSGD^180^ RGAPKRGSGK^190^ DSHTRTTHYG^200^  SLP↓QKSQHGR^210^ TQDENPVVHF^220^ FKNIVTPRTP^230^ PPSQGKGGRD^240^ SRSGSPLEHH^250^  HHHH^254^ | | | |
| Peptide sequences | | Molecular mass, Da | |
|  |  | Calculated | Measured |
| 1 | 117-203 | 9445 | 9456 |
| 2 | 88-126 | 4536 | 4534 |

2)

| MMP-8 fragments | | | |
| --- | --- | --- | --- |
| MGNHSGKREL^10^ SAEKASKDGE^20^ IHRGEAGKKR^30^ SVGKLSQTAS^40^ EDSDVFGEAD^50^  AIQNSGTSAE^60^ DTAVTDSKHT^70^ ADPKN↓NWQGA^80^ HPADPGNRPH^90^ LIRLFSRDAP^100^  GREDNTFKDR^110^ PSESDE↓LQTI^120^ QEDPTAASGG^130^ LDVMASQKRP^140^ SQRS↓KYLATA^150^  STMDHARHGF^160^ LPRHRDTGIL^170^ DSIGRFFSGD^180^ RGAPKRGSGK^190^ DSHTRTTHYG^200^  SLP↓QK↓SQH↓GR^210^ TQDENPVVHF^220^ FKNIVTPRTP^230^ PPSQGKGGRD^240^ SRSGSPLEHH^250^ HHHH^254^ | | | |
| Peptide sequences | | Molecular mass, Da | |
|  |  | Calculated | Measured |
| 1 | 76-205 | 14367 | 14371 |
| 2 | 117-203 | 9445 | 9446 |
| 3 | 145-208 | 7101 | 7096 |

3)

| MMP-9 fragments | | | |
| --- | --- | --- | --- |
| MGNHSGKREL^10^ SAEKASKDGE^20^ IHRGEAGKKR^30^ SVGKLSQTAS^40^ EDSDVFGEAD^50^  AIQNSGTSAE^60^ DTAVTDSKHT^70^ ADPKNNWQGA^80^ HPADPGNRPH^90^ LIRLFSRDAP^100^  GREDNTFKDR^110^ PSESDE↓LQT↓I^120^ QEDPTAASGG^130^ LDVMASQKRP^140^ SQRS↓KY↓LATA↓^150^  STMDHARH↓GF^160^ LPRHRDTGIL^170^ DSIGRFFSGD^180^ RGAPKRGSGK^190^ DSHTRTTHYG^200^  S↓LP↓QK↓SQHGR^210^ TQDENPVVHF^220^ FKNIVTPRTP^230^ PPSQGKGGRD^240^ SRSGSPLEHH^250^  HHHH^254^ | | | |
| Peptide sequences | | Molecular mass, Da | |
|  |  | Calculated | Measured |
| 1 | 1-119 | 12702 | 12703 |
| 2 | 1-116 | 12360 | 12355 |
| 3 | 147-254 | 11958 | 11966 |
| 4 | 159-254 | 10666 | 10664 |
| 5 | 117-203 | 9445 | 9449 |
| 6 | 145-201 | 6282 | 6276 |
| 7 | 151-205 | 6101 | 6094 |

4)

| MMP-10 fragments | | | | | |
| --- | --- | --- | --- | --- | --- |
| MGNHSGKREL^10^ SAEKASKDGE^20^ IHRGEAGKKR^30^ SVGKLSQTAS^40^ EDSDVFGEAD^50^  AIQNSGTSAE^60^ DTAVTDSKHT^70^ ADPKN↓NWQGA^80^ HPADPGNRPH^90^ LIRLFSRDAP^100^  GREDNTFKDR^110^ PSESDE↓LQTI^120^ QEDPTAASGG^130^ LDVMASQKRP^140^ SQRSKYLATA^150^  STMDHARHGF^160^ LPRHRDTGIL^170^ DSIGRFFSGD^180^ RGAPKRGSGK^190^ DSHTRTTHYG^200^  SLP↓QK↓SQHGR^210^ TQDENPVVHF^220^ FKNIVTPRTP^230^ PPSQGKGGRD^240^ SRSGSPLEHH^250^  HHHH^254^ | | | | | |
| Peptide sequences | | Molecular mass, Da | | | |
|  |  | Calculated | | Measured | |
| 1 | 76-205 | | 14367 | | 14371 |
| 2 | 117-203 | | 9445 | | 9446 |

5)

| MMP-12 fragments | | | |
| --- | --- | --- | --- |
| MGNHSGKREL^10^ SAEKASKDGE^20^ IHRGEAGKKR^30^ SVGKLSQTAS^40^ EDSDVFGEAD^50^  AIQNSGTSAE^60^ DTAVTDSKHT^70^ ADPKN↓NWQGA^80^ HPADPGNRPH^90^ LIRLFSRDAP^100^  GREDNTFKDR^110^ PSESDE↓LQTI^120^ QEDPTA↓ASGG^130^ LDVMASQKRP^140^ SQRS↓KYLATA^150^  STMDHARHGF^160^ LPRHRDTGIL^170^ DSIGRFFSGD^180^ RGAPKRGSGK^190^ DSHTRTTHYG^200^  SLP↓QK↓SQH↓GR^210^ TQDENPVVHF^220^ FKNIVTPRTP^230^ PPSQGKGGRD^240^ SRSGSPLEHH^250^  HHHH^254^ | | | |
| Peptide sequences | | Molecular mass, Da | |
|  |  | Calculated | Measured |
| 1 | 76-205 | 14367 | 14371 |
| 2 | 127-254 | 14105 | 14097 |
| 3 | 117-203 | 9445 | 9446 |
| 4 | 145-208 | 7101 | 7096 |

6)

| MT1-MMP fragments | | | |
| --- | --- | --- | --- |
| MGNHSGKREL^10^ SAEKASKDGE^20^ IHRGEAGKKR^30^ SVGK↓LSQTAS^40^ EDSDVFGEAD^50^  AIQNSGTSAE^60^ DTAVTDSKHT^70^ ADPKN↓NWQGA^80^ HPADPGN↓RPH^90^ LIRLFSRDAP^100^  GREDNTFKDR^110^ PSE↓SDE↓LQTI^120^ QEDPTA↓ASGG^130^ LDVMAS↓QKRP^140^ SQRS↓KYLATA↓^150^  STMDHARH↓GF^160^ LPRHRDTGIL^170^ DSIGR↓FFSGD^180^ RGAPKRGSGK^190^ DSHTRTTHYG^200^  S↓LP↓QK↓SQH↓GR^210^ TQDENPVVHF^220^ FKNIVTPRTP^230^ PPSQGKGGRD^240^ SRSGSPLEHH^250^  HHHH^254^ | | | |
| Peptide sequences | | Molecular mass, Da | |
|  |  | Calculated | Measured |
| 1 | 76-205 | 14367 | 14364 |
| 2 | 1-116 | 12360 | 12348 |
| 3 | 117-203 | 9445 | 9447 |
| 4 | 35-113 | 8544 | 8547 |
| 5 | 1-81 | 8387 | 8386 |
| 6 | 145-208 | 7101 | 7098 |
| 7 | 145-201 | 6282 | 6277 |
| 8 | 151-205 | 6101 | 6093 |
| 9 | 117-158 | 4553 | 4553 |
| 10 | 88-126 | 4536 | 4534 |
| 11 | 137-175 | 4459 | 4466 |

7)

| MT2-MMP fragments | | | |
| --- | --- | --- | --- |
| MGNHSGKREL^10^ SAEKASKDGE^20^ IHRGEAGKKR^30^ SVGKLSQTAS^40^ EDSDVFGEAD^50^  AIQNSGTSAE^60^ DTAVTDSKHT^70^ ADPKN↓NWQGA^80^ HPADPGNRPH^90^ LIRLFSRDAP^100^  GREDNTFKDR^110^ PSESDE↓LQTI^120^ QEDPTAASGG^130^ LDVMASQKRP^140^ SQRSKYLATA↓^150^  STMDHARHGF^160^ LPRHRDTGIL^170^ DS↓IGRFFSGD^180^ RGAPKRGSGK^190^ DSHTRTTHYG^200^  SLP↓QK↓SQHGR^210^ TQDENPVVHF^220^ FKNIVTPRTP^230^ PPSQGKGGRD^240^ SRSGSPLEHH^250^  HHHH^254^ | | | |
| Peptide sequences | | Molecular mass, Da | |
|  |  | Calculated | Measured |
| 1 | 76-205 | 14367 | 14367 |
| 2 | 1-116 | 12360 | 12348 |
| 3 | 117-203 | 9445 | 9444 |
| 4 | 117-172 | 6118 | 6113 |
| 5 | 151-205 | 6101 | 6092 |

8)

| MT3-MMP fragments | | | |
| --- | --- | --- | --- |
| MGNHSGKREL^10^ SAEKASKDGE^20^ IHRGEAGKKR^30^ SVGKLSQTAS^40^ EDSDVFGEAD^50^  AIQNSGTSAE^60^ DTAVTDSKHT^70^ ADPKN↓NWQGA^80^ HPADPGNRPH^90^ LIRLFSRDAP^100^  GREDNTFKDR^110^ PSESDE↓LQTI^120^ QEDPTAASGG^130^ LDVMASQKRP^140^ SQRS↓KYLATA↓^150^  STMDHARHGF^160^ LPRHRDTGIL^170^ DSIGRFFSGD^180^ RGAPKRGSGK^190^ DSHTRTTHYG^200^  SLP↓QK↓SQH↓GR^210^ TQDENPVVHF^220^ FKNIVTPRTP^230^ PPSQGKGGRD^240^ SRSGSPLEHH^250^  HHHH^254^ | | | |
| Peptide sequences | | Molecular mass, Da | |
|  |  | Calculated | Measured |
| 1 | 76-205 | 14367 | 14372 |
| 2 | 1-116 | 12360 | 12353 |
| 3 | 117-203 | 9445 | 9448 |
| 4 | 145-208 | 7101 | 7097 |
| 5 | 151-205 | 6101 | 6093 |

9)

| MT4-MMP fragments | | | |
| --- | --- | --- | --- |
| MGNHSGKREL^10^ SAEKASKDGE^20^ IHRGEAGKKR^30^ SVGKLSQTAS^40^ EDSDVFGEAD^50^  AIQNSGTSAE^60^ DTAVTDSKHT^70^ ADPKN↓NWQGA^80^ HPADPGN↓RPH^90^ LIRLFSRDAP^100^  GREDNTFKDR^110^ PSESDE↓LQTI^120^ QEDPTAASGG^130^ LDVMA↓S↓QKRP^140^ SQRS↓KYLATA^150^  STMDHARH↓GF^160^ LPRHRDTGIL^170^ DSIGRFFSGD^180^ RGAPKRGSGK^190^ DSHTRTTHYG^200^  SLP↓QK↓SQH↓GR^210^ TQDENPVVHF^220^ FKNIVTPRTP^230^ PPSQGKGGRD^240^ SRSGSPLEHH^250^  HHHH^254^ | | | |
| Peptide sequences | | Molecular mass, Da | |
|  |  | Calculated | Measured |
| 1 | 76-205 | 14367 | 14364 |
| 2 | 136-254 | 13304 | 13314 |
| 3 | 137-254 | 13217 | 13215 |
| 4 | 1-116 | 12360 | 12347 |
| 5 | 117-203 | 9445 | 9447 |
| 6 | 137-203 | 7460 | 7454 |
| 7 | 145-208 | 7101 | 7096 |
| 8 | 88-136 | 5425 | 5425 |
| 9 | 117-158 | 4553 | 4553 |

10)

| MT6-MMP fragments | | | |
| --- | --- | --- | --- |
| MGNHSGKREL^10^ SAEKASKDGE^20^ IHRGEAGKKR^30^ SVGK↓LSQTAS^40^ EDSDVFGEAD^50^  AIQNSGTSAE^60^ DTAVTDSKHT^70^ ADPKN↓NWQGA^80^ HPADPGNRPH^90^ LIRLFSRDAP^100^  GREDNTFKDR^110^ PSESDE↓LQTI^120^ QEDPTA↓ASGG^130^ LDVMA↓S↓QKRP^140^ SQRS↓KYLATA^150^  STMDHARH↓GF^160^ LPRHRDTGIL^170^ DSIGRFFSGD^180^ RGAPKRGSGK^190^ DSHTRTTHYG^200^  SLP↓QK↓SQH↓GR^210^ TQDENPVVHF^220^ FKNIVTPRTP^230^ PPSQGKGGRD^240^ SRSGSP↓LEHH^250^  HHHH^254^ | | | |
| Peptide sequences | | Molecular mass, Da | |
|  |  | Calculated | Measured |
| 1 | 35-203 | 18302 | 18301 |
| 2 | 76-205 | 14367 | 14368 |
| 3 | 127-254 | 14105 | 14094 |
| 4 | 136-254 | 13304 | 13311 |
| 5 | 137-254 | 13217 | 13218 |
| 6 | 1-116 | 12360 | 12350 |
| 7 | 159-246 | 9602 | 9599 |
| 8 | 117-203 | 9445 | 9447 |
| 9 | 145-208 | 7101 | 7098 |
